# Environmental Lead Exposure and Adult Literacy in Myanmar: An Exploratory Study of Potential Associations at the Township Level

**DOI:** 10.3390/ijerph15061086

**Published:** 2018-05-28

**Authors:** Robert C. MacTavish, Liam W. Rémillard, Colleen M. Davison

**Affiliations:** 1 Department of Environmental Sciences, University of Guelph, 50 Stone Road East, Guelph, ON N1G 2W1, Canada; mactavir@uoguelph.ca; 2 Department of Public Health Sciences, Queen’s University, 99 University Ave, Kingston, ON K7L 3N6, Canada; 11lr13@queensu.ca

**Keywords:** lead exposure, adult literacy, global health, environmental health, Myanmar

## Abstract

Environmental lead exposure is a population health concern in many low- and middle-income countries. Lead is found throughout Myanmar and prior to the 1940s the country was the largest producer of lead worldwide. The aim of this study was to examine any potential association between lead mining and adult literacy rates at the level of 330 townships in Myanmar. Townships were identified as lead or non-lead mining areas and 2015 census data were examined with association being identified using descriptive, analytical and spatial statistical methods. Overall, there does appear to be a significant relationship between lead mining activity and adult literacy levels among townships with both low access (*p* = 0.05; OR = 2.701 (1.136–6.421)) as well with high access to safe sanitation (*p* = 0.01; OR = 18.40 (1.794–188.745)). Local Indicators of Spatial Association (LISA) cluster maps confirm these findings. This exploratory analysis is a first step in the examination of potential environmental lead exposure and its implications in Myanmar.

## 1. Introduction

Literacy, traditionally defined as proficiency in reading and writing, is an important determinant of health and can support an individual’s functional role in society [1]. Adult literacy has been used as one marker of cognitive proficiency and intelligence [2], as well as being a facilitator of comprehension and reasoning [3]. Illiteracy in adult populations may be attributed to many variables, including limited access to educational institutions or opportunities due to low socioeconomic status [4,5]. Exposure to harmful environmental contaminants has also been adversely associated with cognitive function and literacy. Lead exposure has specifically been associated with negative outcomes related to neurological function, cognitive proficiency, intelligence quotient (IQ) and literacy [3].

The most common pathway of lead toxification is through inhalation of lead via the respiratory tract; however, the heavy metal can also be absorbed in the gastrointestinal tract via ingestion [6]. Lead can then be redistributed to the skeletal system, where it may have a half-life of 5 to 19 years [6,7]. The main anatomical target during lead absorption and toxification is the nervous system, which can lead to adverse neurological functions such as ataxia, coma, or even death [6]. Additional neurological impacts include reductions in attention span and levels of educational engagement, leading to a subsequent decrease in educational attainment [5]. Furthermore, heavy metals such as lead have a high affinity and can bind to *N*-methyl-d-aspartate receptors found in nerve cells, resulting in cognitive dysfunction due to reactive oxygen species [8].

There are no previous studies specifically looking at the cognitive outcome of adult literacy in relation to lead exposure at a population level. However, one previous study does conclude that people residing in lead mining communities have a higher exposure and greater absorbance of lead than populations residing in areas without lead mining activity [9]. This increase in lead absorption could be detrimental to human health and development, as prolonged exposure may result in the neurological impacts mentioned.

Myanmar (previously known as Burma), is a country with a large mining industry and great potential for further mine development, as its lands are rich with jade, oil and metals [10,11]. Lead has great historical significance in the country. Prior to the Second World War, Myanmar was the world’s largest producer of lead, providing a significant source of economic activity [11]. Today, following recent political and economic reform, many lead mines in Myanmar are developed through foreign investment as well as Myanmar’s Ministry of Mines [11]. However, it is recognized that private investors, small-scale artisan extraction, and informal or undocumented groups also conduct lead mining. Despite the potential economic benefits, there are many negative human health implications of lead toxification, particularly regarding impacts upon human brain development and neurological function [3,4,6]. To date, there is no research evidence available related to the level of environmental contamination or the potential health effects of lead exposure for populations in Myanmar. However, studies conducted on Burmese refugee populations in the United States indicate that blood lead levels may be elevated in this population [12]. In addition, although there is no available data on blood lead concentrations for Myanmar’s populations, particularly those residing near lead mining developments, past research has concluded that people residing near lead mines in Klity Village, Thailand demonstrated a significant inverse relationship between IQ levels and both tooth lead and blood lead concentrations [13]. As this village is located near Myanmar’s borders, it is possible that populations residing near lead mines in Myanmar may be similarly impacted.

In this exploratory study, townships in Myanmar were categorized as “lead” or “non-lead” mining, and census data for adult literacy levels and socioeconomic levels were examined. Myanmar’s country-wide adult literacy rate was reported as 89.5%; this cognitive-related indicator has also been determined at the township level in the 2014 Myanmar Census [14]. This study specifically explored the relationship between lead exposure and adult literacy level, based on residency in townships of lead mining. This relationship may be modified by the level of affluence or poverty. To explore this effect, a classical, stratified analysis was undertaken. Indicators of Myanmar’s economy, as collected from the 2014 Census, including unemployment rates, the poverty gap index, food poverty percentages, and employment rates were available at the state but not the township level. Therefore, access to safe sanitation was selected as a proxy measure for affluence as it was an indicator available at the township level. As there is minimal research regarding the human health impact of lead mining in Myanmar, this study may reveal potential population-level health and mining exposure patterns that could be further investigated in future field-based studies.

## 2. Materials and Methods

The main research question for this study was whether adult literacy levels differed across lead mining and non-lead mining townships in Myanmar with low and high levels of access to safe sanitation. To answer this question, our research objectives were:

(1) To screen and categorize the 330 townships in Myanmar as either lead mining or non-lead mining;
(2) to use the 2014 Myanmar Census data to describe and map the adult literacy level and level of access to safe sanitation (as a measure of poverty/affluence) at the township level;
(3) to stratify the townships by low and high levels of access to improved sanitation and to examine the relationship between lead exposure and Myanmar’s adult literacy in these two strata;
(4) to explore global and local tests of spatial autocorrelation to identify patterns of clustering; and to map and report the results to inform future studies.

We began by establishing a series of study hypotheses. First, that adult literacy levels will be lower in lead mining townships than in townships without lead mining. It is assumed this relationship is due to the negative effect of lead exposure upon neurological and cognitive development of which adult literacy is a proxy measure; second, that poverty or affluence levels modify the relationship between lead mining exposure and adult literacy; third that access to safe sanitation is a proxy measure for poverty/affluence that has a relevant threshold in Myanmar; and finally fourth that townships with low adult literacy levels may cluster in similar locations to lead mining townships.

### 2.1. Data Collection

Data for the outcome (adult literacy) and development indicator (access to safe sanitation) were available in Myanmar’s 2014 Census, which was undertaken by the Ministry of Immigration and Population and supported by the United Nations Development Program (UNDP). Data collected from the country-wide census was accessed through the Myanmar Information Management Unit (MIMU). The 2014 Myanmar Census was the first census conducted in Myanmar since 1983, following the transition of Burma to Myanmar [14]. Receiving a vast amount of international support, the census results included over 50 million Burmese citizens. The adult literacy levels, and levels of access to safe sanitation as an indicator of socioeconomic status were collected at a township level.

Locations of lead deposits in Myanmar are well documented and tend to be concentrated in specific areas including the Shan State and the Kayah State [11]. Lead mining takes place at large and small scales in the country, however there are no complete and current maps of lead mining activity in Myanmar. The specific location of lead mining sites, and thus the lead mining townships, were identified using three sources of information. First, coordinates for lead mines were recorded using yearly reports from the United States Geological Survey (USGS) for Myanmar. In addition, socio-economic analyses and occupational reports from MIMU were used to corroborate and add to the USGS records of lead mining activity. The mineralogy database, Mindat, was used to identify and confirm mining sites across Myanmar. It should be noted that these data sources comprehensively captured large- and medium-scale mining activities; however, it is possible that very small-scale lead mines went undetected.

### 2.2. Study Population

Myanmar has a population of approximately 51.5 million people located in 14 state regions, subdivided further into 330 townships [14]. As stated previously, Myanmar’s lead mining development could have critical implications for human health and development, particularly in lead mining areas. For instance, the Hpasaung Township in the Kayah State is known to have a large portion of its population working in lead mines. Citizens in this township have expressed concern about water pollution from lead mine development, indicating worry for adequate safety regulations [15]. Improper safety precautions in regions of lead mining development could not only provide a hazardous occupational exposure towards workers in the mining industry, but also could impact the surrounding township through lead dust or contamination of sediments and water sources.

### 2.3. Study Design

The first portion of this exploratory cross-sectional study was a descriptive analysis. Townships were identified as a lead mining township if there was a confirmed active lead mine, or if there had been a mine within the last 50 years, as lead can leech into soils and continuously contaminate the surrounding groundwater long after such mining sites close [16]. Townships were also considered as lead mining if there was an active lead mine within 15 km of its borders, as research indicates that communities living several kilometers from lead mines still have a significant exposure to lead when compared to the general population [9]. Prevalence of illiteracy and lead mining in Myanmar were described using counts and proportions and visually assessed using GeoDa mapping software (v1.12) (Luc Anselin, Chicago, United States of America) and Geographic Information System (GIS) base-maps (Myanmar Information Management Unit, Yangon, Myanmar) with the 330 townships delineated.

To assess the degree of patterning in the data, techniques of exploratory spatial data analysis were applied. First, a global indicator of spatial autocorrelation—Moran’s I—was applied to the adult literacy, safe sanitation, and lead mining variables [17,18]. Spatial autocorrelation is a measure of how similar one value is to its neighboring values [19]. Moran’s I is measured from negative one to positive one with values further from zero indicating decreasing spatial randomness, whereby a value of positive one indicates perfect clustering, and a value of negative one indicates perfect dispersion [19]. Although Moran’s I provides insight towards the global spatial patterns, this method is not able to identify cluster locations or the type of clustering. Local Indicators of Spatial Association (LISA) was applied to each of the three variables to identify the presence of localized clusters [20]. LISA is a spatial analytic tool adapted from the Moran’s I and conducts spatial autocorrelation test statistics at a localized level for each unit of geography to identify statistically significant clusters. To verify the significance of the LISA cluster maps, 999 random Monte Carlo permutations were calculated. The LISA maps characterize five different types of spatial relationships: (1) High-High, indicating clustering of high values surrounded by high values; (2) High-Low, indicating high values surrounded by low values; (3) Low-Low, indicating clustering of low values surrounded by low values; (4) Low-High, indicating low values surrounded by high values; and (5) Not-Significant, indicating the absence of spatial autocorrelation [20]. 

To conduct global and local tests of spatial autocorrelation, a spatial weights matrix had to be specified [20,21]. The purpose of the spatial weights matrix is to identify the extent to which neighboring geographic units are associated with each other. This study utilized a first-order queen contiguity spatial weights matrix, whereby a geographic unit will only be affected by the immediately contiguous units sharing a common border or vertex.

Following the descriptive analysis, the relationship between lead exposure and adult literacy was assessed using a stratified, classical analysis where the primary exposure was lead mining in Myanmar’s townships and the primary outcome was low adult literacy levels. Townships were categorized as having high adult literacy levels if over 89.1% of their adult population was literate. This was chosen as the cutoff for the dichotomy as it included the upper two tertiles and had practical relevance. Townships were categorized as having high access to safe sanitation if over 83.3% of their population had access to safe sanitation, also representing the upper two tertiles. Statistical differences between the stratified odds ratios were determined using the Mantel-Hantzel chi-square test for homogeneity. Analyses were conducted using SPSS (v.24) (IBM, Armonk, NY, USA) and Stata (v15.1) (Microsoft, Redmond, WA, USA).

## 3. Results

### 3.1. Demographic Description

The demographic description of Myanmar’s population (Table 1) indicates that there are discrepancies between Myanmar’s townships in terms of both access to safe sanitation and adult literacy levels.

The proportion of the population with access to safe sanitation across Myanmar townships ranges from 5.4% to 100.0%. It was reported that many urban centers have relatively high levels of access to safe sanitation, while many rural areas are not able to afford infrastructure for improved sanitation [22]. Similarly, the range of adult literacy levels among townships was large (11.5–99.2%). The Yangon State had the highest adult literacy rates (96.6%), while the Shan State had the lowest adult literacy rates (64.6%) [14,23]. Alongside the low adult literacy levels, the Shan State also had much of the lead mining activity in Myanmar, with 17 lead mining townships. It is worth noting that the Shan State also had generally low levels of access to safe sanitation (average of 56.75%), indicating that it may be a state of low affluence or more limited development.

**Figure 1:**
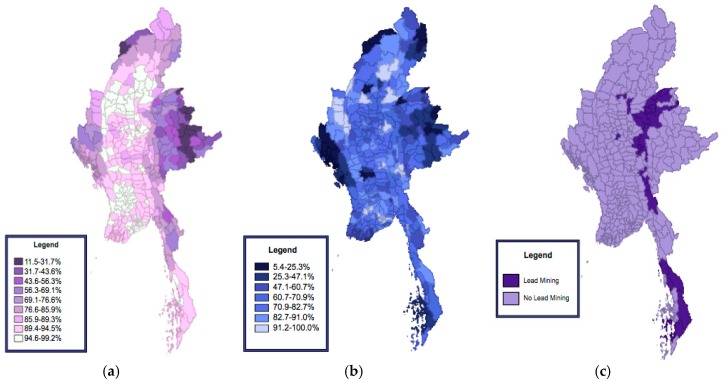
Descriptive maps of Myanmar visually representing adult literacy levels (**a**), access to safe sanitation (**b**), and lead mining activity status (**c**). Both access to safe sanitation and adult literacy levels were mapped using natural breaks calculated through GeoDa, while the lead mining map represents dichotomous variables.

**Figure 2:**
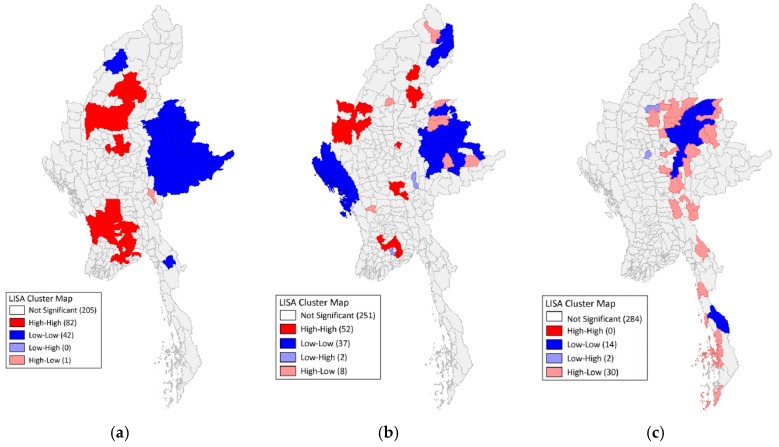
(**a**) Local Indicators of Spatial Association (LISA) Cluster Map of Adult Literacy at the Geographic Level of Myanmar Townships. (**b**) LISA Cluster Map of Access to Safe Sanitation at the Geographic Level of Myanmar Townships. (**c**) LISA Cluster Map of Lead Mining at the Geographic Level of Myanmar Townships.

The demographic descriptions of adult literacy levels, access to safe sanitation, and lead mining are represented in Figure 1, while the LISA cluster maps for each of the respective variables are represented in Figure 2.

### 3.2. Adult Literacy

When exploring the choropleth map of adult literacy in Figure 1a, it appears that there is a high concentration of low adult literacy townships in the Eastern regions of Myanmar, followed by smaller aggregations in the Western and Northern regions. Further analysis with the Moran’s I also suggests a strong degree of positive spatial autocorrelation with a test statistic of 0.83. When exploring the LISA map of adult literacy in Figure 2a, it is apparent that there are statistically significant “Low-Low” clusters of low adult literacy predominantly in the Eastern and Western regions of Myanmar previously identified in the choropleth maps of Figure 1, and two distinct “High-High” clusters of high adult literacy in central Myanmar.

### 3.3. Access to Safe Sanitation

Like the spatial patterns depicted by adult literacy rates, there appears to be a concentration of townships with low access to safe sanitation in most Eastern, Western, and Northern regions of Myanmar as depicted in Figure 2b. A Moran’s I statistic of 0.53 suggests that access to safe sanitation exerts moderate positive spatial autocorrelation, albeit less than what was depicted with adult literacy. When exploring the LISA map of access to safe sanitation in Figure 2b, three “Low-Low” clusters of poor access to safe sanitation emerge in the areas identified by the choropleth maps, as well as 5–6 “High-High” clusters in central Myanmar. 

### 3.4. Lead Mining

The lead mining choropleth map depicted in Figure 2c differs from the previous two figures in that there appears to be a narrow ribbon of lead mining townships from central to southern Myanmar. The Moran’s I statistic of 0.38 indicates a low degree of positive spatial autocorrelation and suggests that lead mining is relatively diffused in Myanmar. The findings from the LISA cluster map in Figure 2c parallel that of the Moran’s I since the LISA map uncovered two localized clusters of lead mining townships in central and southern Myanmar. 

### 3.5. Cluster Location Comparisons

Based on the exploratory spatial data analysis, it reveals there is likely a spatial correlation between adult literacy and access to safe sanitation, based on the location of the statistically significant “Low-Low” cluster in eastern Myanmar. In addition, it appears there could be a relationship between lead mining activity and low adult literacy levels due to overlapping geographic units depicted in the central Myanmar clusters for both variables. These descriptive assessments were further investigated during the statistical analysis.

### 3.6. Lead Mining and Adult Literacy

There is a significant relationship between lead mining activity and adult literacy levels among townships with low access to safe sanitation (*p* = 0.05; OR = 2.701 (1.136–6.421)) as well with high access to safe sanitation (*p* = 0.01; OR = 18.40 (1.794–188.745)) (Table 2). There was a meaningful and statistically significant difference between the stratum-specific odds ratios (*p* < 0.001). Due to small cell sizes in the contingency tables, however, there were wide confidence intervals and thus the results must be assessed with caution. Additional covariates could be assessed for potential confounding if more township level data becomes available for analyses in Myanmar in the future.

## 4. Discussion

Lead mining activity in Myanmar tends to be localized in certain spatial areas, such as the Kayah, Shan and Tanintharyi States, all regions of large lead deposits [10]. Spatial and statistical analyses reveal significant associations between lead mining and adult literacy at the township level, with differential risks in areas of low and high access to safe sanitation, our chosen development or affluence indicator. Many areas of low access to safe sanitation also had low levels of adult literacy, and we acknowledge the well-established relationship between poverty and adult literacy [24,25,26]. However, our findings indicate that citizens living in townships with high access to safe sanitation and lead mining activity may also be more at risk of having lower adult literacy than those in similar situations but in non-mining townships. Further exploration of this pattern is warranted particularly at community and individual levels. Do citizens in these townships have high lead levels in biological samples?

Myanmar citizens located in proximity to lead mines could be exposed to lead from contaminated soils, lead dust, or ingesting contaminated drinking water or food items [27,28]. As many people living in lead mining townships do not have adequate access to safe drinking water or sanitation, this could increase their consumption of lead due to contaminated water sources [15,23], while it is also possible that bottled water is used. Lower adult literacy levels in lead mining townships could be due to lead’s ability to impact neurological functions [5]. It is expected that there is an increased exposure to environmental lead for children in lead mining townships. For instance, dust lead levels have been found to be significantly associated with blood lead levels in children whose specific behavioral and physiological characteristics make them particularly at risk to environmental exposures [29,30]. It is likely that Myanmar citizens located in lead mining townships could have increased lead toxification if exposed to lead from an early age. Additionally, it was reported in one study that children from low-income families are at a differential risk for being exposed to environmental lead, highlighting ongoing environmental injustices occurring today [31]. It is possible that lower-income families have resided near lead mining activities for a lack of a better option, demonstrating the relationship between lead mining activity and affluence. The collection and testing of biological specimens from residents in different townships could confirm or refute these ideas although currently there is limited opportunity to conduct these kinds of studies in Myanmar (personal communication, WHO-Myanmar Country Representative Dr. Jorge Luna, 2 February 2016).

Early exposure to environmental lead could have severe and long-lasting impacts upon cognitive function, causing cognitive impairment that lasts well into adulthood. It has been determined that along with hypochromic anemia and lead-caused encephalopathy, high lead exposure has also resulted in poorer school performance, a decreased IQ and hyperactivity [32]. Many studies have reported a positive association between lead exposure and encephalopathy [32,33,34]. Lead encephalopathy may abrupt neurological function, as it is associated with difficulties in concentrating, behavioral problems and restlessness. In more severe cases, it may cause confusion, difficulty in understanding and deterioration of memory storage. Understandably, the occurrence of encephalopathy should be further explored, as alongside other cognitive dysfunctions, it could have resulted in increased illiteracy rates found in Myanmar’s lead mining townships.

As mentioned previously, many studies have looked at the association between lead exposure and reduced IQ [3,27,35,36]. It has been reported that alongside a lower IQ, high lead exposure can also impact emotional well-being, aggression and anxiety [35]. These negative impacts on emotional health could contribute to poor performance in academic settings and potential illiteracy. Although adult literacy has not been used as an indicator of cognitive function in studies of lead exposure, there are studies indicating that IQ and memory are significantly associated with performance in adult literacy tests [37]. A reduction in IQ from lead toxification could therefore contribute to decreased adult literacy levels in lead mining townships.

Although children are arguably at a much greater risk of the acute and chronic impacts of environmental lead exposure, lead may also have direct detrimental consequences upon the cognitive functioning of adults. Therefore, it is also possible that short-term residents of lead mining townships in Myanmar could also have had neurological impacts, decreasing their literacy capability. For instance, lead accumulation in adults may result in increased brain lesions, and a subsequent decline in cognitive function [38]. This decrease in cognitive function could result in reduced verbal and visual memory, declining verbal learning and a decrease in actual brain size from cell death. In fact, epidemiological studies have indicated that lead exposure may increase the rate of cognitive decline in older populations, as increased blood lead concentrations were associated with lower scores on a mental status examination [39]. The neurodegenerative effects of lead exposure have also been studied, and it has been reported that increased lead exposure is significantly associated with Parkinson’s Disease, amyotrophic lateral sclerosis, and potentially Alzheimer’s Disease [40,41,42]. Therefore, it is possible that even individuals without early age exposure to lead could encounter cognitive deficits when moving to a lead mining township in Myanmar. This could reduce the literacy capability in Myanmar’s adult population.

This exploratory study fills an important gap in literature that could help inform future etiological studies about the negative health effects of lead exposure from mining development in Myanmar. As previous studies have not investigated lead mining in Myanmar, this is the first study to categorize and map Myanmar’s townships as lead or non-lead mining. There has also been an absence of studies exploring lead’s impact upon adult literacy as an indicator of cognitive function. Additionally, the 2014 Myanmar Census was the first undertaken in 30 years and provides extensive data for most Myanmar’s population. Along with other reports from MIMU, the USGS, and mineralogy databases, this study draws information from high-quality and current data sources. The study plan and results were discussed with public health stakeholders in Myanmar to ensure the appropriateness of the design, analysis and dissemination of findings. The World Health Organization (WHO) outlined that lead mining in lower-income countries, including Myanmar, is a grave concern because of possible adverse effects during brain development [43].

There are many strengths of this study, however there are also important limitations to consider. As mentioned, even though Myanmar’s larger lead mining development areas are identified, it is possible that smaller-scale productions could go unnoticed. Small and independent operations may not necessarily report lead mining activity to Myanmar’s government [10,15]. In addition, lead can travel in water, air and soil, and is present in leaded gasoline, paints and other products. We recognize that lead exposure could extend beyond the immediate zones around mining sites. This kind of more extended assessment of broader environmental exposure will not be possible to capture in this study and this limitation is recognized. Although adults readily absorb lead, or may have absorbed lead when they were children, direct measures of cognitive development among younger populations would have been an ideal addition to this study. Unfortunately, there are no township-specific data sets available for cognitive or neurological indicators for children in Myanmar. Lead exposure is cumulative and affects neurological function over time, so if adults had moved in and out of lead mining areas this may represent a limitation to the study. Migration between townships was not available in the census data, and therefore could not be incorporated into the results. Moreover, there is a potential for ecological fallacy in this study, as data in the Census was not available at a finer-scale than at the township level. It is unlikely that information at the township level can account for individual citizen diversity within a township. In addition to the ecologic fallacy, there is potential for reverse causality given the use of cross-sectional data sources, it is possible that people with lower literacy levels gravitate to lead mining areas. The safe sanitation indicator is also a limited proxy measure for level of affluence. Ideally, as additional environmental and township level data becomes available in Myanmar, fulsome epidemiological regression analyses will be possible. Given the limitations mentioned, we propose this study as the first exploratory step in what might represent a longer program of research. The current study has successfully tested an approach to spatial and population health analysis that could be used to examine other potential environmental health concerns, particularly in areas where field study might be difficult. These kinds of studies, while inherently limited, represent starting points in examining potential health or illness threats. Results from this study are being used to advocate for further focused environmental health studies in Myanmar.

## 5. Conclusions

With growing development of lead mining projects in Myanmar [10] and other low- and middle-income countries, these results signify the importance of further investigation and the implementation of safety measures to guard against harmful exposure. It is essential to test and regulate lead concentrations found in soil and water sources and to protect workers in lead mines to prevent lead poisoning and toxification [27]. Ongoing surveillance should be explored in current lead mining operations, while site-specific remediation plans should be implemented on inactive lead mining sites.

Overall, there was a significant relationship calculated between lead mining and adult literacy at the township level in Myanmar. Future studies should measure the extent of human and environmental lead accumulation in lead mining and neighboring areas. Lead exposure, health and cognitive function research should be conducted at the individual level. In addition, further studies should investigate the role of lead exposure in the cognitive development of Myanmar’s children.

## Figures and Tables

**Table 1:** Descriptive Characteristics of the Study Sample (Townships in Myanmar, *n* = 330).

Descriptor	Townships in Myanmar *n* (%)
**Lead Mine Development**	
Lead mining activity	29 (8.79%)
No lead mining activity	301 (91.21%)
**Adult Literacy**	
High adult literacy level (>95% of township population) ^1^	105 (31.82%)
Low adult literacy level (<95% of township population)	225 (68.18%)
**Access to Safe Sanitation**	
Access for >90% of township population ^2^	63 (19.09%)
Access for 70–90% of township population	148 (44.85%)
Access for <70% of township population	120 (36.6%)
**Average and Range of Descriptors at the Township Level**	**Percentage (%)**
Average adult literacy rate	85.56%
Range of adult literacy rates	11.5–99.2%
Average level of access to safe sanitation	71.42%
Range of access to safe sanitation	5.4–100.0%

^1^ The United Nations Educational, Scientific and Cultural Organization (UNESCO) reported that there was a National Goal to achieve 95.0% adult literacy levels in Myanmar by 2010 [22]. ^2^ The national strategy for the Government of Myanmar is to achieve 90% of the population having access to safe sanitation within the next 10 years [23].

**Table 2:** Cross Tabulation of Lead Mining Activity and Adult Literacy in Myanmar’s Townships, and the Effect of Access to Safe Sanitation on This Relationship (*n* = 330).

Descriptor	Low Adult Literacy Level (≤89.1% of Population)	High Adult Literacy Level (>89.1% of Population)	*p*-Value
**Lead Mining Development**			
Lead Mining	19 (5.76%)	10 (3.03%)	<0.01 *
No Lead Mining	92 (27.88%)	209 (63.33%)	
**Access to Safe Sanitation**			
High Proportion (>83.3% of Population)	18 (5.45%)	93 (28.18%)	<0.01 *
Low Proportion (<83.3% of Population)	93 (28.18%)	126 (38.18%)	
**Townships with High Access to Safe Sanitation (>83.3%)**			
Lead Mining	3 (0.91%)	1 (0.30%)	0.01 **
No Lead Mining	15 (4.55%)	92 (27.88%)	
**Townships with Low Access to Safe Sanitation (<83.3%)**			
Lead Mining	16 (4.85%)	9 (2.73%)	<0.05 *
No Lead Mining	77 (23.33%)	117 (35.45%)	

* *p*-value from Chi-Square test (one-tailed), with significance indicated when *p* < 0.05. ** *p*-value from Fisher’s Exact Test (one-tailed) with significance indicated when *p* < 0.05.

## References

[1] White S., McCloskey M. (2003). Framework for the 2003 National Assessment of Adult Literacy. U.S. Department of Education.

[2] OECD/Statistics Canada (2005). Learning a Living: First Results of the Adult Literacy and Life Skills Survey.

[3] Carlisle J.C., Dowling K.C., Siegel D.M., Alexeff G.V. (2009). A blood lead benchmark for assessing risks from childhood lead exposure. J. Environ. Sci. Health A.

[4] Von Stumm S., Plomin R. (2015). Socioeconomic status and the growth of intelligence from infancy through adolescence. Intelligence.

[5] Lind A., Johnston A. (1990). Adult Literacy in the Third World: A Review of Objectives and Strategies.

[6] Katzung B.G., Masters S.B., Trevor A.J. (2012). Heavy metal intoxication & chelators. Basic & Clinical Pharmacology.

[7] Rabinowitz M.B. (1991). Toxicokinetics of bone lead. Environ. Health Perspect..

[8] Karri V., Schuhmacher M., Kumar V. (2016). Heavy metals (Pb, Cd, As and MeHg) as risk factors for cognitive dysfunction: A general review of metal mixture mechanism in brain. Environ. Toxicol. Pharmacol..

[9] Grigoryan R., Petrosyan V., Melkom Melkomian D., Khachadourian V., McCartor A., Crape B. (2016). Risk factors for children’s blood lead levels in metal mining and smelting communities in Armenia: A cross-sectional study. BMC Public Health.

[10] Reynolds N. (2015). Myanmar’s Mining Industry.

[11] Gardiner N.J., Robb L.J., Searle M.P. (2014). The metallogenic provinces of Myanmar. Appl. Earth Sci..

[12] Mitchell T., Jentes E., Ortega L., Sucosky M.S., Jefferies T., Bajcevic P., Parr V., Jones W., Brown M.J., Painter J. (2012). Lead poisoning in United States-bound refugee children: Thailand-Burma border, 2009. Pediatrics.

[13] Pusapukdepob J., Sawangwong P., Pulket C., Satraphat D., Saowakontha S., Panutrakul S. (2007). Health risk assessment of villagers who live near a lead mining area: A case study of Klity Village, Kanchanaburi Province, Thailand. Southeast Asian J. Trop. Med. Public Health.

[14] Myanmar Information Management Unit (MIMU) Population in General. http://www.themimu.info/Census_2014_SR_dashboard%20.

[15] Myanmar Information Management Unit (MIMU) Kayah State Socio-Economic Analysis. http://www.themimu.info/sites/themimu.info/files/documents/Asses-sment_Ka-yah_State_Socio-Economic_Analysis_-_Annexes_EU_Oct2013.pdf.

[16] Kwak J., Kim K., Park M., Kim J., Park K. (2012). Determination of lead in soil at a historical mining and smelting site under laser-induced breakdown spectroscopy. Environ. Technol..

[17] Anselin L. (2008). Spatial Econometrics: Methods and Models.

[18] Moran P. (1948). The interpretation of statistical maps. J. R. Stat. Soc..

[19] Getis A. (2008). A history of the concept of spatial autocorrelation: A geographer’s perspective. Geogr. Anal..

[20] Anselin L. (1995). Local Indicators of Spatial Association—LISA. Geogr. Anal..

[21] Getis A., Aldstadt J., Anselin L., Ray S. (2010). Perspectives on Spatial Data Analysis.

[22] Myanmar Information Management Unit National Strategy for Rural Water Supply, Sanitation and Hygiene (WASH). http://www.themimu.info/sites/themimu.info/files/docume-nts/National_Strategy_for_Rural_Water_Supply_Sanitation_Hygiene_WASH_2016-2030_ENG.pdf.

[23] UNESCO The Government of the Republic of the Union of Myanmar Ministry of Education: National Education for All (EFA) Review Report. http://unesdoc.unesco.org/images/0022/-002297/229723E.pdf.

[24] Kirsch I.S. (1993). Adult Literacy in America: A First Look at the Results of the National Adult Literacy Survey.

[25] Kutner M., Greenberg E., Jin Y., Boyle B., Hsu Y., Dunleavy E. (2003). Literacy in Everyday Life: Results from the 2003 National Assessment of Adult Literacy.

[26] Agneta L., Anton J. (1990). Adult Literacy in the Third World: A Review of Objectives and Strategies. https://eric.ed.gov/?id=ED339819.

[27] Pekar Z., Riley N. (2006). Lead Human Exposure and Health Risk Assessments and Ecological Risk Assessment for Selected Areas: External Review Draft Technical Report.

[28] Maddaloni M., Bellew M., Diamond G., Follansbee M., Gefell D., Goodrum P., Johnson M., Koporec K., Khoury G., Luey J. (2005). Assessing lead risks at non-residential hazardous waste sites. Hum. Ecol. Risk Assess..

[29] Lanphear B.P., Roghmann K.J. (1997). Pathways of lead exposure in urban children. Environ. Res..

[30] Harvey P.G., Spurgeon A., Morgan G., Chance J., Moss E. A method for assessing hand-to-mouth activity in children as a possible transport route for toxic substances. Proceedings of the 5th International Conference on Heavy Metals in the Environment.

[31] Malcoe L.H., Lynch R.A., Keger M.C., Skaggs V.J. (2002). Lead sources, behaviours, and socioeconomic factors in relation to blood lead of native American and white children: A community-based assessment of a former mining area. Environ. Health Perspect..

[32] Papanikolaou N.C., Hatzidaki E.G., Belivanis S., Tzanakakis G.N., Tsatsakis A.M. (2005). Lead toxicity update: A brief review. Med. Sci. Monit..

[33] Jarup L. (2003). Hazards of heavy metal contamination. Br. Med. Bull..

[34] Chisolm J.J. (2001). The road to primary prevention of lead toxicity in children. Pediatrics.

[35] Chen A., Cai B., Dietrich K.N., Radcliffe J., Rogan W.J. (2007). Lead exposure, IQ, and behavior in urban 5- to 7-year olds: Does lead affect behavior only by lowering IQ?. Pediatrics.

[36] McMichael A.J., Baghurst P.A., Vimpani G.V., Wigg N.R., Robertson E.F., Tong S. (1994). Tooth lead levels and IQ in school-age children: The Port Pine cohort study. Am. J. Epidemiol..

[37] Alloway T.P., Gregory D. (2013). The predictive ability of IQ and working memory scores in literacy in an adult population. Int. J. Educ. Res..

[38] Stewart W.F., Schwartz B.S., Davatzikos C., Shen D., Liu D., Wu X., Todd A.C., Shi W., Bassett S., Youssem D. (2006). Past adult lead exposure is linked to neurodegeneration measured by brain MRI. Neurology.

[39] Wright R.O., Tsaih S.W., Schwartz J. (2003). Lead exposure biomarkers and mini-mental status exam scores in older men. Epidemiology.

[40] Weisskopf M.G., Weuve J., Nie H., Saint-Hilaire M.H., Sudarsky L., Simon D.K., Hersh B., Schwartz J., Wright R.O., Hu H. (2010). Association of cumulative lead exposure with Parksinson’s Disease. Environ. Health Perspect..

[41] Kamel F., Umbach D.M., Hu H., Munsat T.L., Shefner J.M., Taylor J.A., Sandler D.P. (2005). Lead exposure as a risk factor for amyotrophic lateral sclerosis. Neurodegener. Dis..

[42] Bakulski K.M., Rozek L.S., Dolinoy D.C., Paulson H.L., Hu H. (2012). Alzheimer’s Disease and environmental exposure to lead: The epidemiologic evidence and potential role of epigenetics. Curr. Alzheimer Res..

[43] Tong S., Schirnding Y.E., Prapamontol T. (2000). Environmental lead exposure: A public health problem of global dimensions. Bull. World Health Organ..

